# Supplementary material for: Disentangling climate and policy uncertainties for the Colorado River post-2026 operations
Source: Nat Commun. 2025 Sep 29;16:8625. doi: 10.1038/s41467-025-63635-4 (PMC12479936; doi:10.1038/s41467-025-63635-4)
Supplement: Supplementary file 1 — Supplementary Information [file 41467_2025_63635_MOESM1_ESM.pdf]

Disentangling Climate and Policy Uncertainties for the Colorado River Post-2026 Operations

Supplementary Materials

Bowen Wang[1,2]*, Benjamin Bass[1]*, Alex Hall[1], Stefan Rahimi[1,3], Lei Huang[1]

[1] Department of Atmospheric and Oceanic Sciences, University of California, Los Angeles, Los Angeles, CA, USA.

[2] Department of Civil and Environmental Engineering, Massachusetts Institute of Technology, Cambridge, MA, USA.

[3] Department of Atmospheric Science, University of Wyoming, Laramie, WY, USA.

*Corresponding Author: Bowen Wang (bowenwang23@g.ucla.edu); Benjamin Bass (benb0228@g.ucla.edu)

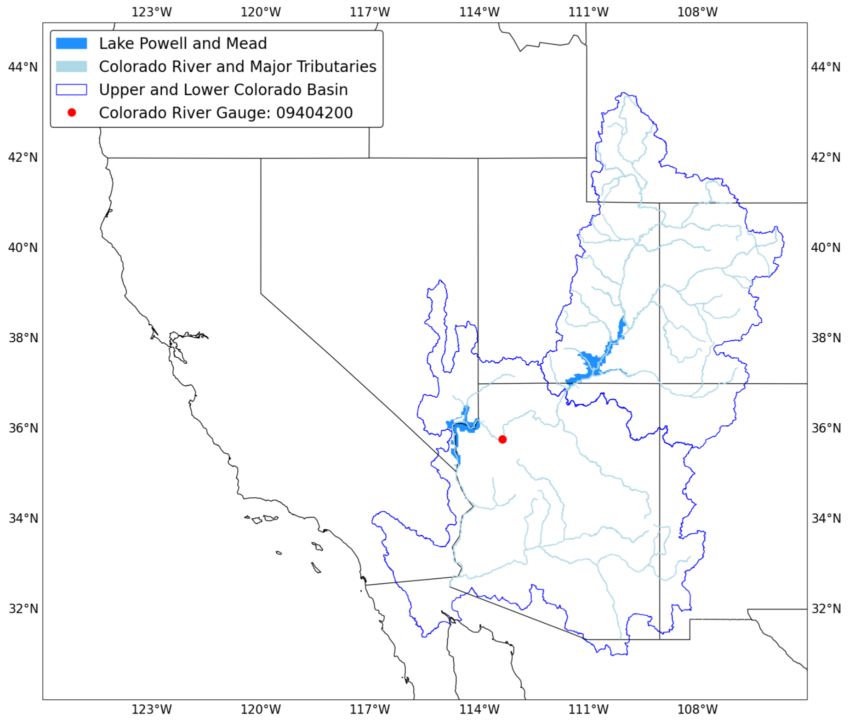

Supplementary Figure 1. Study area of the Colorado River Basin (CRB). Streamflow in the Upper Colorado River Basin (UCRB) represents the majority of the basin's total streamflow and is what drives the water budget model (WBM).

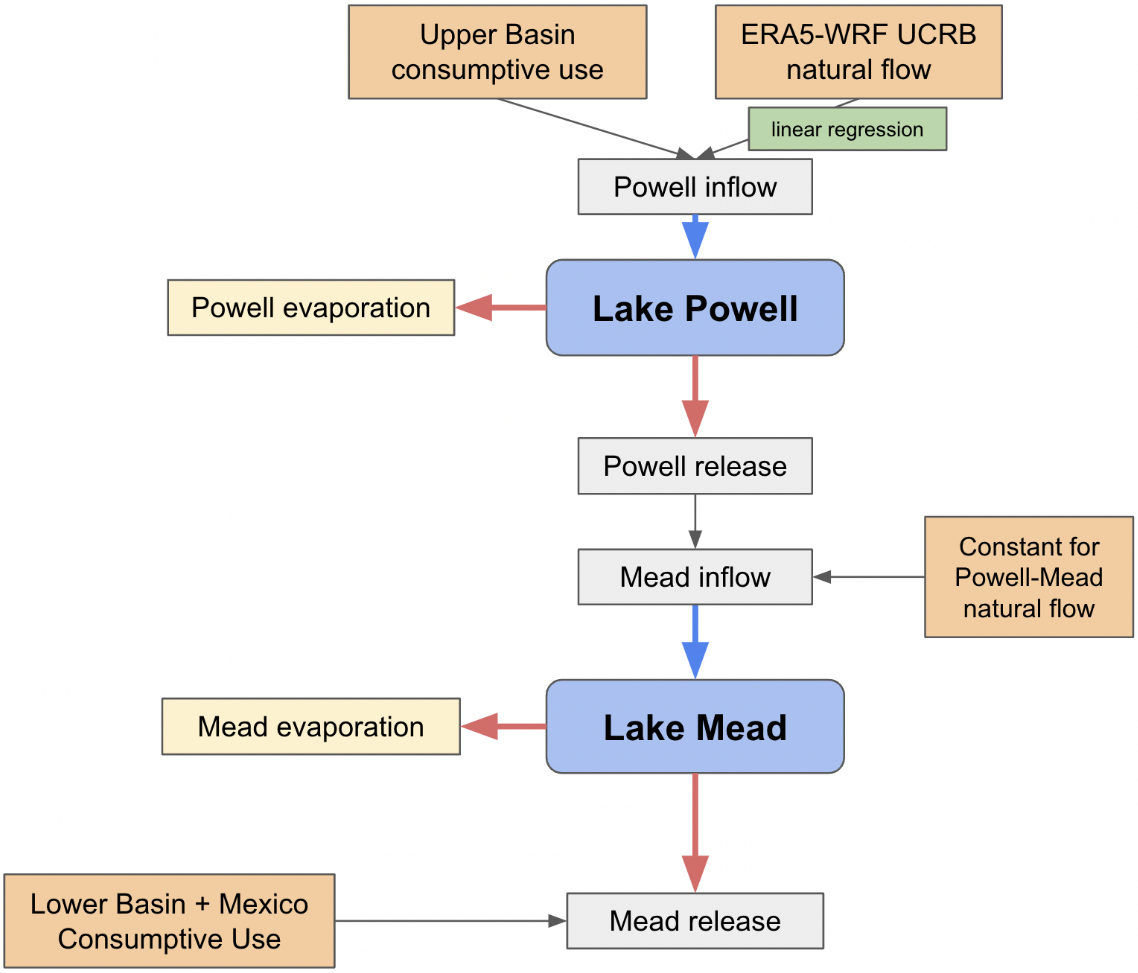

Supplementary Figure 2. Schematic of the water budget model (WBM). Blue arrows represent inflows into a reservoir, while red arrows represent outflows (release or evaporation). Black arrows represent a statistical (linear) or policy-based relationship between the linked variables.

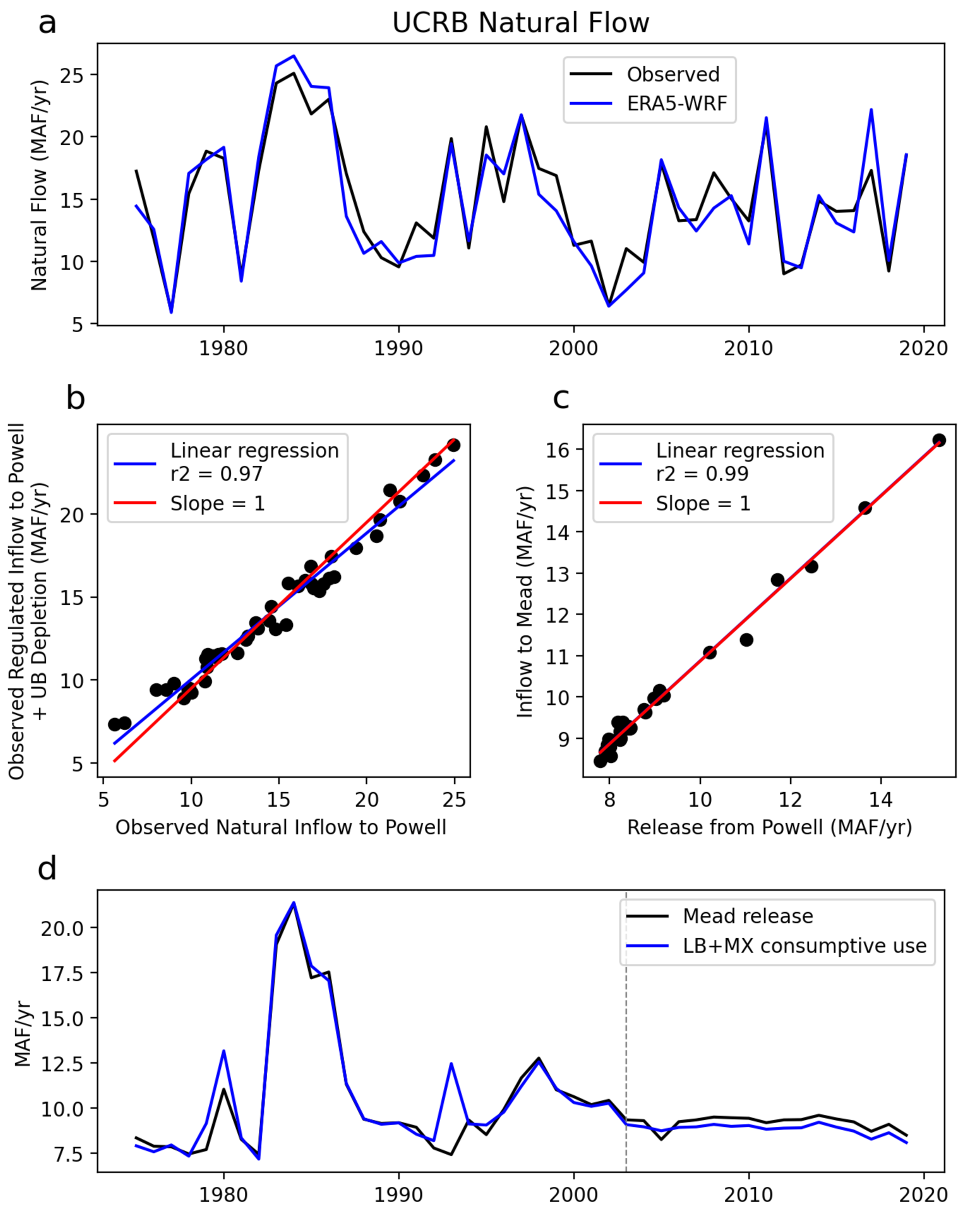

Supplementary Figure 3. **Validation of each model component.** **(a)** Comparison between observed and modeled UCRB natural flow (ERA5-WRF; Bass et al., 2023a; Bass et al., 2023b), **(b)** linear regression between observed natural and regulated inflow to Powell, **(c)** linear regression between observed release from Powell and regulated inflow to Mead at the Colorado River above Diamond Creek gauge (site number 09420400), and **(d)** comparison between the combined consumptive uses of Lower Basin states and Mexico and the release from Mead.

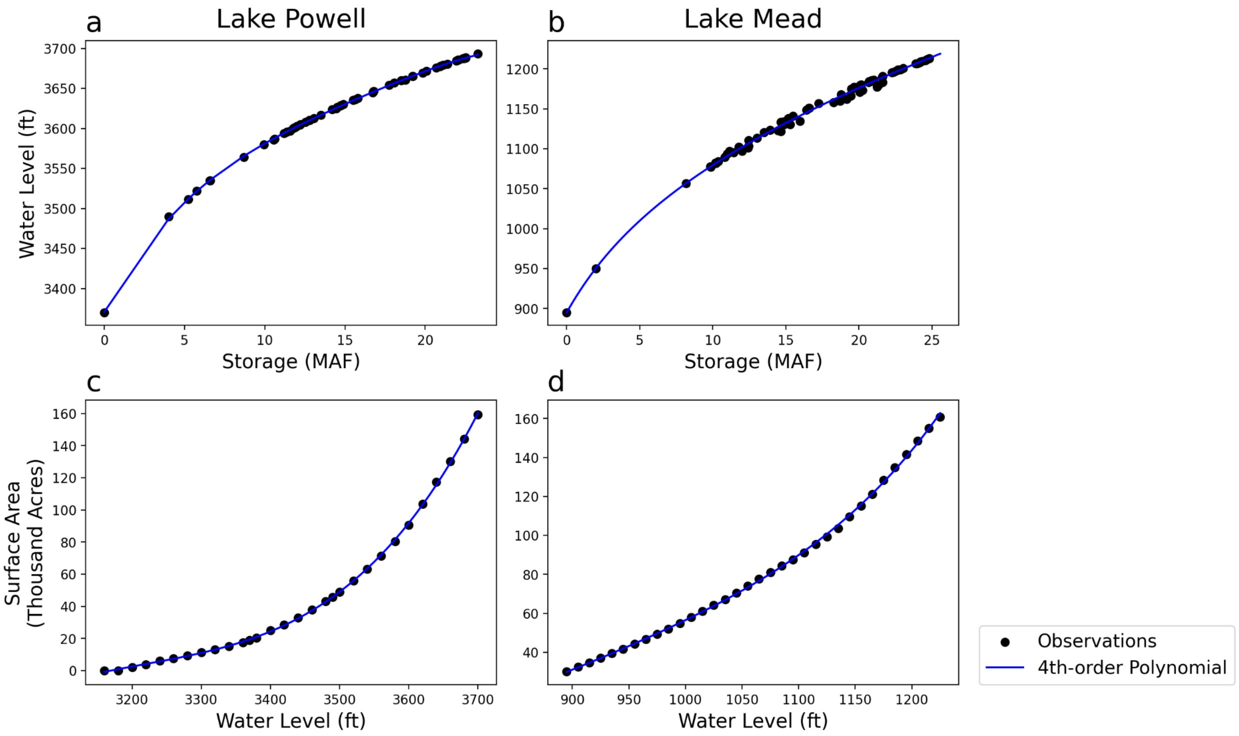

Supplementary Figure 4. Empirical relationships between reservoir water levels and storage. 4th-order polynomials used to calculate **(a,b)** water level from storage and **(c,d)** surface area from water levels for Lakes **(a,c)** Powell and **(b,d)** Mead.

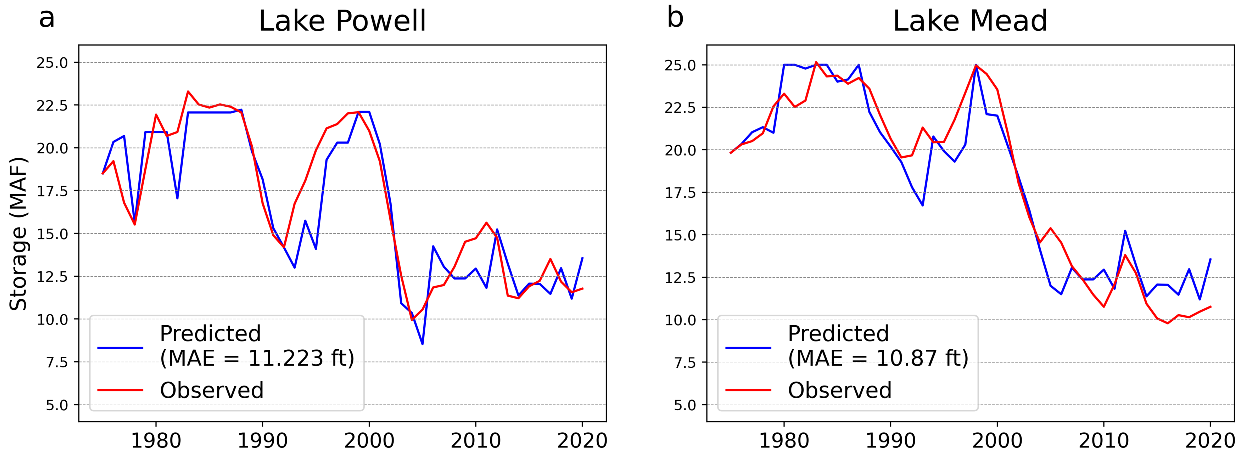

Supplementary Figure 5. **Evaluation of WBM performance.** Evaluation of WBM's prediction of water levels in the historical period against the observed water levels for Lakes **(a)** Powell and **(b)** Mead.

Supplementary Table 1. The Interim Guidelines of 2007 (IG2007) water operation guidelines for Lake Powell. The guidelines explain how Lake Powell should release water given each water level condition. *This value, known as the equalization elevation, is set to increase gradually from 3636 to 3666 ft between 2008 and 2026. It is assumed to be 3666 ft thereafter.

**Lake Powell water level (ft)**	**Interim Guidelines 2007**<br>(for DCP2019<br>and DCP2019+)
**3700 - 3666***	If Powell is expected to spill, release to keep it within capacity;<br>Balance storage between Mead and Powell;<br>or release 8.23 MAF
**3666* - 3575**	Release 8.23 MAF<br>If Mead < 1075ft: balance storage between Mead and Powell, but release between 7.0 and 9.0 MAF
**3575 – 3525**	If Mead ≥ 1025ft: release 7.48 MAF<br>If Mead < 1025ft: release 8.23 MAF
**3525 – 3370**	Balance storage between Mead and Powell, but release between 7.0 and 9.5 MAF

Supplementary Table 2. The DCP2019 and DCP2019+ delivery curtailment schemes for Lake Mead. The DCP2019 and DCP2019+ schemes decide delivery curtailments based on Lake Mead water levels. Reductions are made from the total allocation of 9 million acre-feet (MAF) for the Lower Basin (CA, AZ, NV, and Mexico). All values below are in thousand acre-feet (TAF).

Lake Mead water level (ft)	DCP2019 (thousand acre-ft)	DCP2019+ (thousand acre-ft)
**1,090 – >1,075**	241	441
**1,075 – 1,050**	613	1146
**<1,050 – >1,045**	721	1338
**1,045 – >1,040**	1013	1984.4
**1,040 – >1,035**	1071	2237
**1,035 – >1,030**	1129	2412
**1,030 – 1,025**	1188	2671
**<1,025 – 1,000**	1375	3275
**<1,000 – 975**	1375	3608
**<975 – 950**	1375	3942
**<950**	1375	4275

Supplementary Table 3. **The dynamically downscaled GCM ensemble.** The downscaled GCMs and respective members used in this study, and the linear trends (from 1984 to 2099) in temperature, precipitation, and runoff associated with each GCM member.

**GCM**	**Member**	Temperature (°C/decade)	Precipitation (%/decade)	Runoff (%/decade)
ACCESS-CM2	r5i1p1f1	0.66	0.61	-1.01
CanESM5	r1i1p2f1	0.77	-0.35	-3.44
CESM2	r11i1p1f1	0.49	0.50	-1.40
CNRM-ESM2-1	r1i1p1f2	0.51	-0.36	-2.95
EC-Earth3	r1i1p1f1	0.61	-0.62	-4.59
EC-Earth3-Veg	r1i1p1f1	0.58	-0.95	-3.83
MIROC6	r1i1p1f1	0.48	-0.66	-3.52
MPI-ESM1-2-HR	r7i1p1f1	0.46	0.26	-1.32
NorESM2-MM	r1i1p1f1	0.46	-0.44	-1.70
TaiESM1	r1i1p1f1	0.64	-0.15	-2.28
Ensemble mean		0.57	-0.22	-2.61

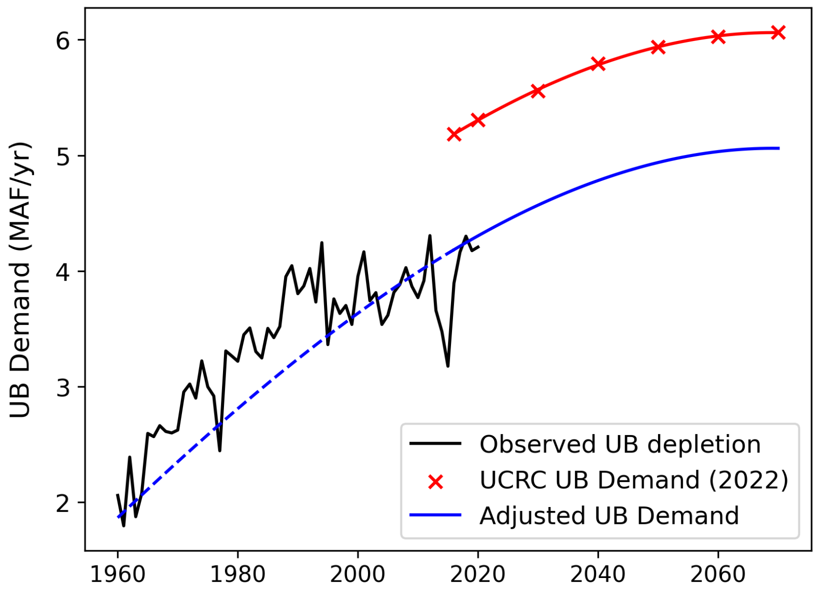

Supplementary Figure 6. Future projections for UB demand. Red crosses show the Upper Colorado River Commission (UCRC) projections at 5-year intervals, and the red curve is a 3rd-order polynomial fit to the projections. The UCRC projections (blue) were adjusted to match the observed UB depletion records (black).

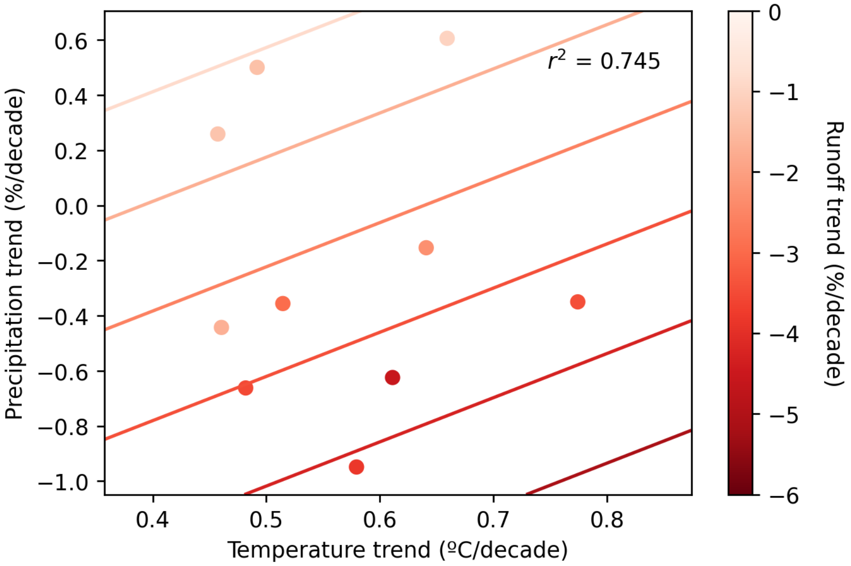

Supplementary Figure 7. GCM runoff sensitivities to temperature and precipitation. The GCM-projected runoff trends in the UCRB are primarily influenced by the precipitation trends of the respective GCMs (p = 0.002), while the temperature trends also play an important (yet weaker and statistically insignificant) role (p = 0.154).

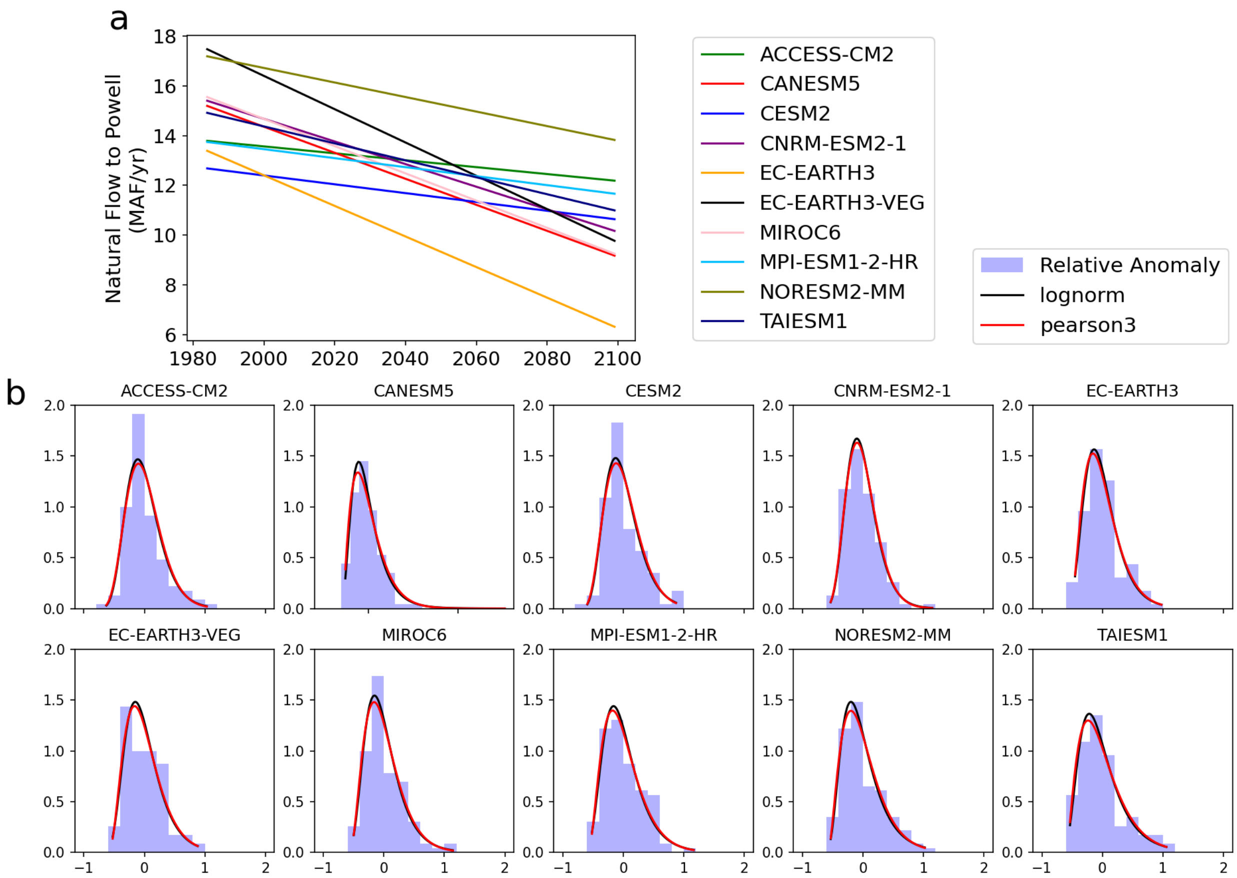

Supplementary Figure 8. **GCM runoff trends and distributions of variability.** (**a**) Linear trends obtained from the 10 GCM runoff time-series and (**b**) their detrended annual fractional anomalies unexplained by AR(1), and the probability distribution functions fit by the Pearson-III and lognorm distributions.

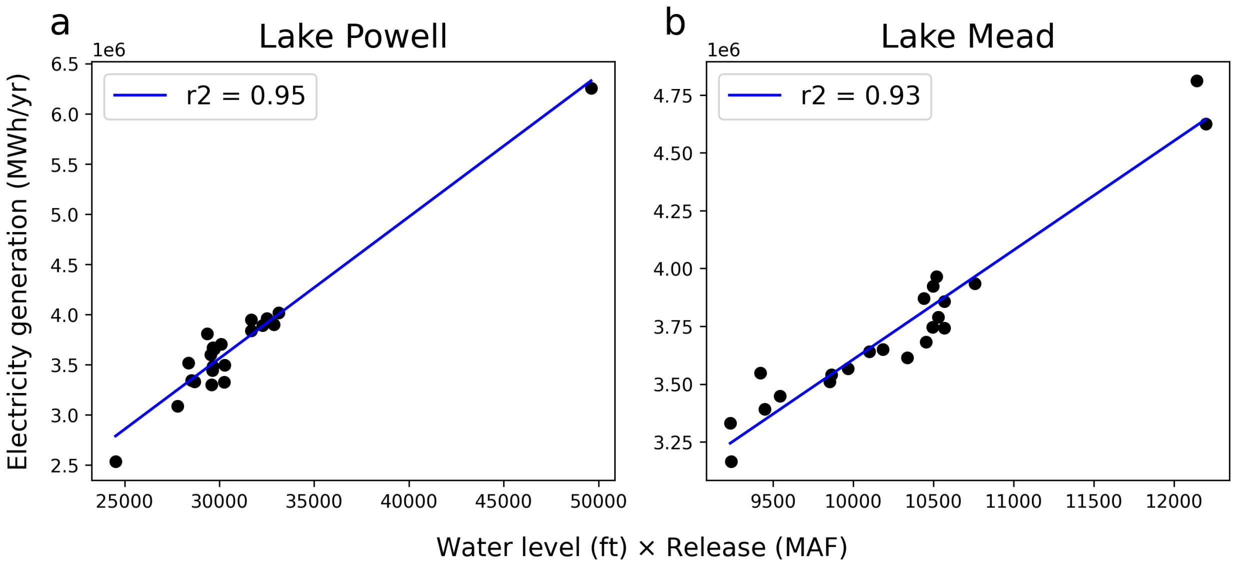

Supplementary Figure 9. **Empirical relationships for predicting hydropower generation.** Linear regressions used to translate the water levels and reservoir release to hydroelectric generation for Lakes **(a)** Powell and **(b)** Mead.

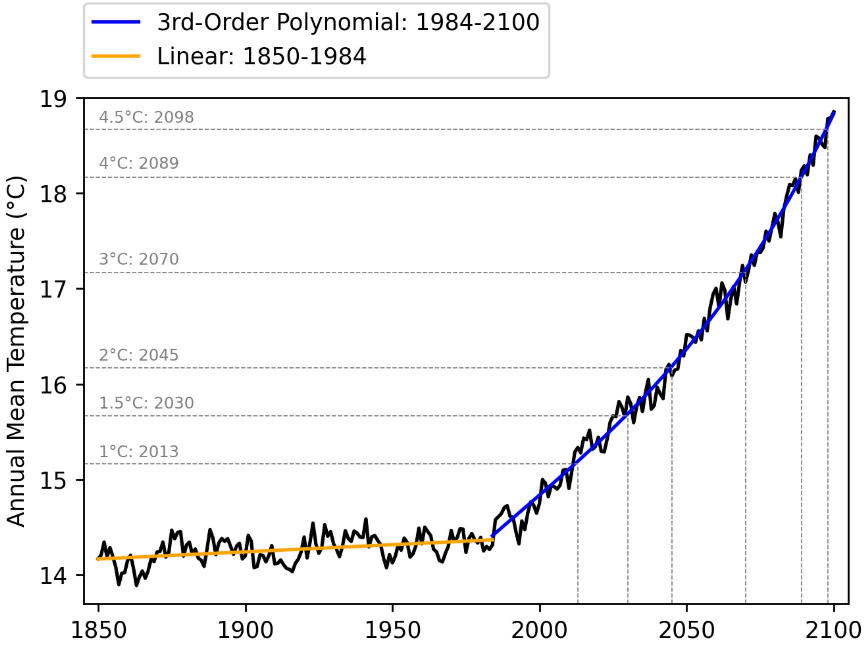

Supplementary Figure 10. **Trend of GCM-based global warming levels.** Linear regression (1850-1984) and 3rd-order polynomial (1984-2100) used to derive global warming levels. Time-series is based on the 10 GCM ensemble mean of global (non-downscaled) temperature under the SSP3-7.0 emission trajectory. Important warming levels and the years when they are reached are noted.

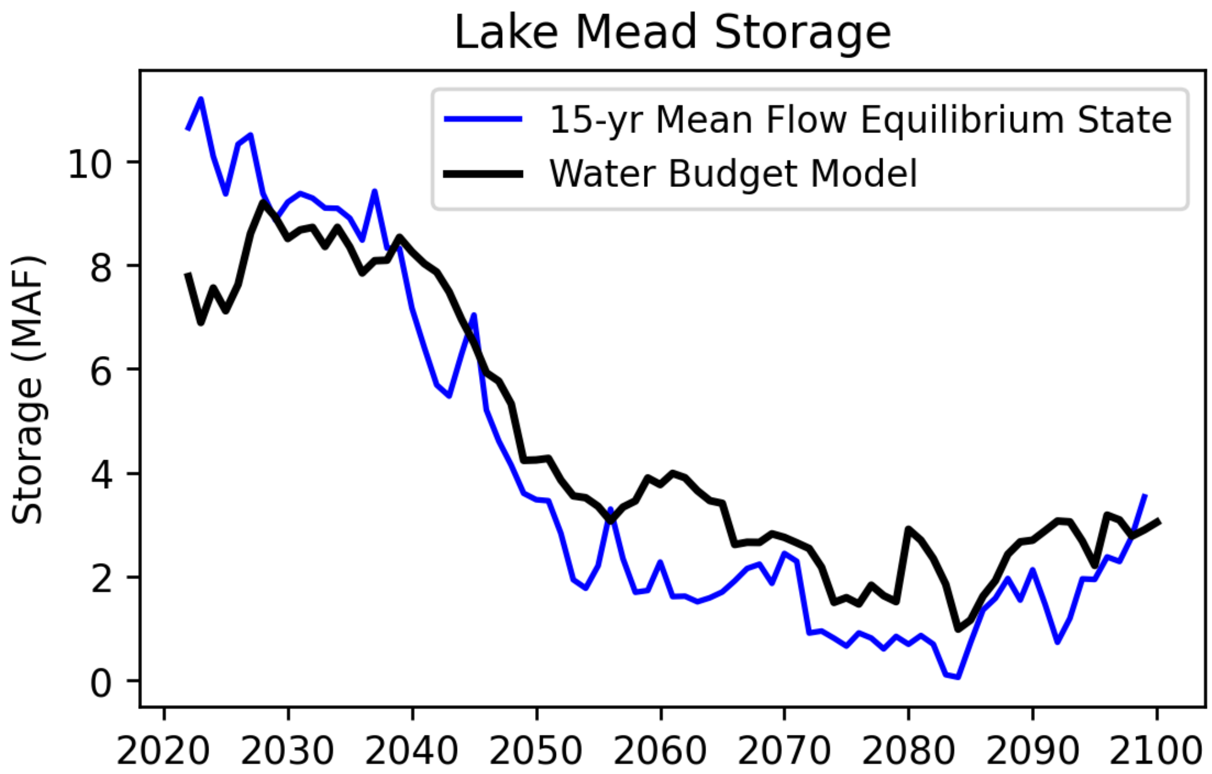

Supplementary Figure 11. Equilibrium water levels produced from 15-year mean streamflow can be used to reproduce WBM projections of Lake Mead. Black line shows the mean projection of the 10-member dynamically downscaled ensemble. Values in the blue line are instead the GCM ensemble mean from running the equilibrium state simulation using the 15-year mean streamflow preceding the projected year from each GCM as the steady flow input. This indicates that the reservoir has a roughly 15-year memory of the natural streamflow conditions.

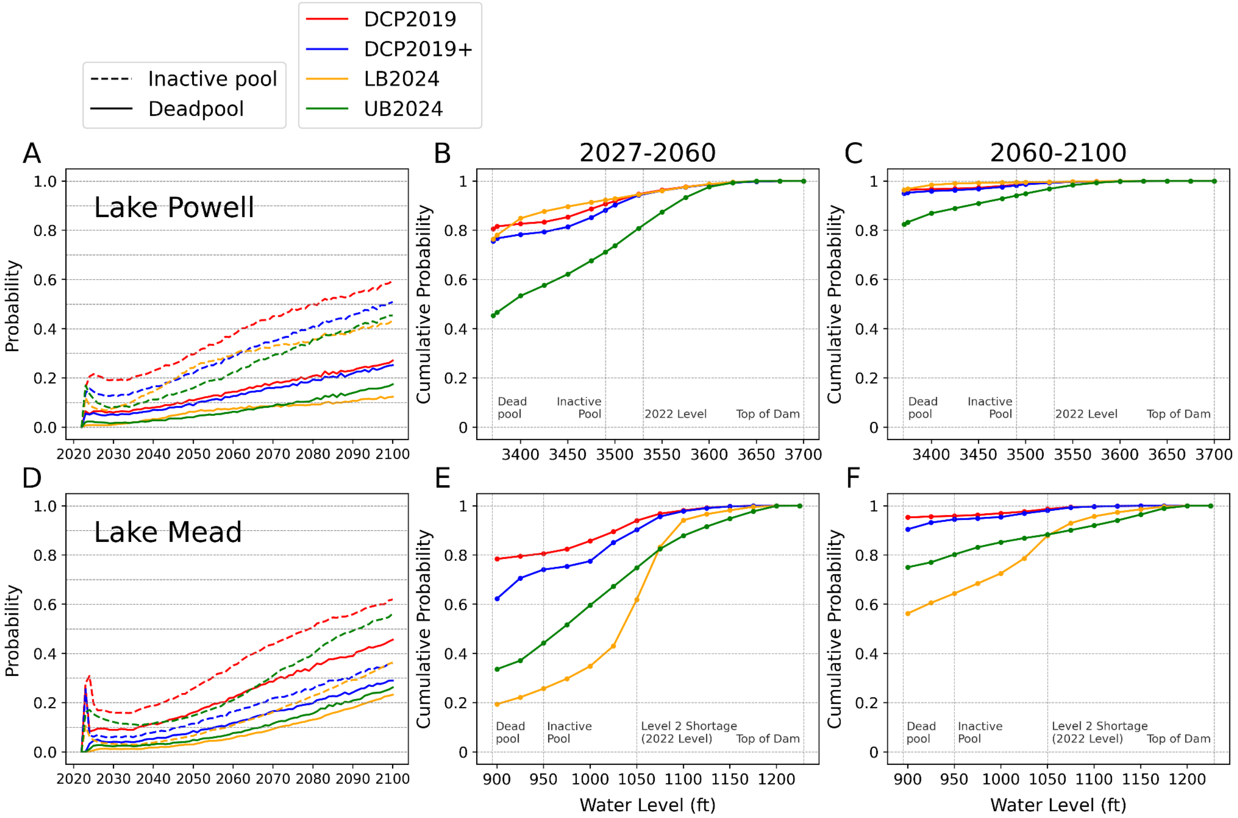

Supplementary Figure 12. Sensitivity of projected reservoir risks to model setup. Same as Figure 2, but using equation 3, which removes the linear regression between UCRB natural flow minus UB consumption and the regulated inflow into Lake Powell.

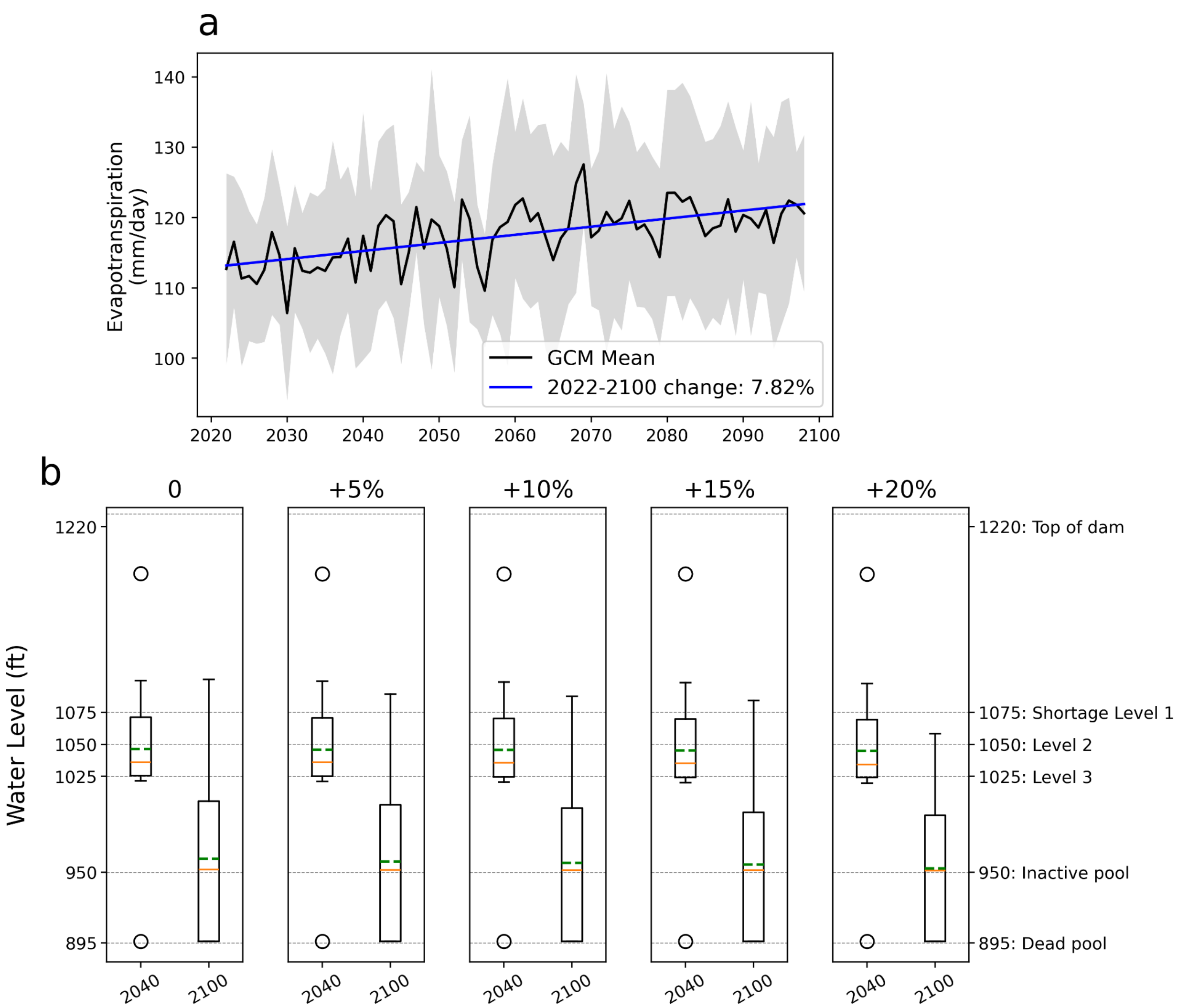

Supplementary Figure 13. **Sensitivity of projected reservoir risks to lake evaporation parameters.** **(a)** Projected change in evapotranspiration in the UCRB between 2022 and 2100 based on the downscaled GCM ensemble. Grey shadings indicate one standard deviation around the ensemble mean. **(b)** Distribution of Lake Mead mid-century (left boxplots) and end-of-century (right boxplots) water level projections based on 5 different trends in evaporation at Powell and Mead, using the 10 dynamically downscaled GCM streamflow series. The middle panel is the same as the boxplot in **Fig. 1d**. The evaporation rate constants (ft/year, **Equations 5 and 6**) change linearly from 2022 to 2100 by a rate such that it will change by the labeled percent value by 2100. Water levels generally decrease as evaporation rates increase, but the impact tends to be negligible, particularly for the 7.8% evaporation increase observed by the ensemble mean trend. In **(b)**, the boxes represent the interquartile ranges (IQR). The solid orange line represents the median of the ensemble, and the dashed green line is the mean. The whiskers extend to the first/last data point whose distance to Q1/Q3 is within 1.5 times the IQR, and the individual points are beyond the range of the whiskers.

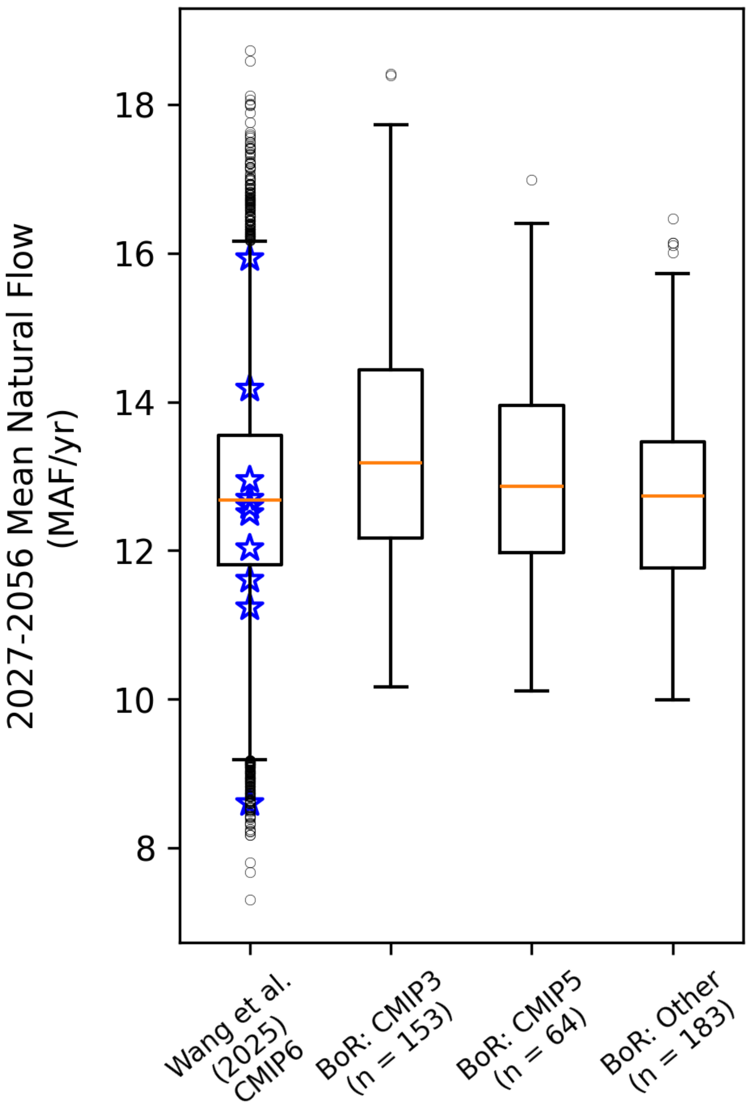

Supplementary Figure 14. **Comparison of hydrology scenarios in this study and those of Bureau of Reclamation.** Comparison between the natural flow conditions in the hydrology ensemble used in this study with ensembles developed by the Bureau of Reclamation for the 2027-2056 period. Blue stars in the left column show the 10 dynamically downscaled GCM members, and the boxplot shows the distribution of the 10,000 time series developed using the Monte Carlo approach. The three boxplots on the right show natural flow conditions from the Bureau of Reclamation's CMIP3, CMIP5, and non-CMIP-based ensembles.
